# Combinational Analyses with Multiple Methods Reveal the Existence of Several Forms of Polysialylated Neural Cell Adhesion Molecule in Mouse Developing Brains

**DOI:** 10.3390/ijms21165892

**Published:** 2020-08-16

**Authors:** Airi Mori, Yi Yang, Yuka Takahashi, Masaya Hane, Ken Kitajima, Chihiro Sato

**Affiliations:** 1 Bioscience and Biotechnology Center, Nagoya University, Chikusa, Nagoya 464-8601, Japan; mori.airi@d.mbox.nagoya-u.ac.jp (A.M.); yi.yang@jt.com (Y.Y.); takahashi.yuka@i.mbox.nagoya-u.ac.jp (Y.T.); mhane@agr.nagoya-u.ac.jp (M.H.); kitajima@agr.nagoya-u.ac.jp (K.K.); 2 Graduate School of Bioagricultural Science, Nagoya University, Chikusa, Nagoya 464-8601, Japan; 3 Institute for Glyco-Core Research (iGCORE), Nagoya University, Chikusa, Nagoya 464-8601, Japan

**Keywords:** polysialic acid, neural cell adhesion molecule (NCAM), development, native PAGE, complex formation

## Abstract

Polysialic acid (polySia/PSA) is an anionic glycan polymer of sialic acid, and it mostly modifies the neural cell adhesion molecule (NCAM) in mammalian brains. Quality and quantity of the polySia of the polySia–NCAM is spatio-temporally regulated in normal brain development and functions, and their impairments are reported to be related to diseases, such as psychiatric disorders and cancers. Therefore, precise understanding of the state of polySia–NCAM structure would lead to the diagnosis of diseases for which their suitable evaluation methods are necessary. In this study, to develop these evaluation methods, structures of polySia–NCAM from mouse brains at six different developmental stages were analyzed by several conventional and newly developed methods. Integrated results of these experiments clearly demonstrated the existence of different types of polySia–NCAMs in developing brains. In addition, combinational analyses were shown to be useful for precise understanding of the quantity and quality of polySia, which can provide criteria for the diagnosis of diseases.

## 1. Introduction

Polysialic acid (polySia/PSA) is a linear polymer of sialic acid (Sia) with a degree of polymerization (DP) of 8–400 Sia residues [1]. In nature, polySia was first discovered in bacterial glycocalyx (*Escherichia coli* K1 and *Neisseria meningitidis* group B) [2,3] and later in vertebrates; polysialoglycoproteins in fish eggs [4] and a neural cell adhesion molecule (NCAM) in the brain [5] were demonstrated to have polySia structure. The majority of polySia (90~96.5%) was shown to link to NCAM in the brain, based on the results using NCAM-knockout (KO) mice [6,7]; however, other molecules, such as a sodium channel α subunit [8] and SynCAM (CADM1) [7], are also known to share the rest of polySia in the brain. The presence of CD36 in milk [9], Neuropillin-2 (NRP2) [10], and CCR7 [11] are also known to contain polySia in other tissues than the brain. In case of microglia, NCAM was firstly demonstrated to be polysialylated [12], and then using microglia cell line and THP-1 macrophage cells, the presences of polySia in E-selectin-ligand 1 and NRP2 were also reported [13]. The polysialyltransferases also seem to have ability to synthesize polySia onto own N-glycans, although the natural occurrence and the biological significance is not known in detail [14]. As the expression of polySia is highly restricted in embryonic brains and almost all polySia disappears in the adult brain, the spatio-temporal modification of polySia toward NCAM is an important feature for normal brain development [15]. Recently, the expression of polySia has been demonstrated to continue, especially in the olfactory system and hippocampus, where neurogenesis is ongoing, even in adult brains [16,17]. In addition, the presence of polySia-expressing cells in the amygdala, prefrontal cortex, and hypothalamus have also been demonstrated [18,19], although the precise function in each area in the adult brain has not yet been well-studied.

The function of polySia is thought to be an anti-adhesive effect toward molecules, membranes, and cells, because polySia has a large exclusion volume [15]. The homophilic and heterophilic bindings of NCAMs and other cell adhesion molecules, such as TAG-1, the heparan sulfate proteoglycan (HSPG), the chondroitin sulfate proteoglycan (CSPG), and the fibroblast growth factor receptor (FGFR), regulate their signaling via molecule, cell, and membrane interactions [15]. Therefore, polySia is considered to inhibit their bindings and be a regulator for the cell–cell interface. Through its anti-adhesive effect, polySia is considered to be involved in the organized brain development [15]. The polySia-impaired mice, NCAM-KO mice [6], polySia-biosynthesizing enzyme [20], ST8 alpha-*N*-acetyl-neuraminide alpha-2,8-sialyltransferase 2 (St8sia2)-KO mice [21], and ST8 alpha-*N*-acetyl-neuraminide alpha-2,8-sialyltransferase 4 (St8sia4)-KO mice [22] showed abnormal memory, circadian rhythm, and behavior, supporting that polySia expression is highly regulated for organized brain structure [23]. Interestingly, polySia also has an attractive property toward neurologically active molecules, such as the brain-derived neurotrophic factor (BDNF) [24], FGF2 [25], and dopamine [26], which are very important for neural development and brain functions. By reserving such molecules, polySia regulates their functions [19]. When polySia is expressed in heparan sulfate (HS)-expressing cells, FGF2-driven cell proliferation via the FGF2-HS-FGFR complex is inhibited, indicating that polySia is a primary reservoir of this molecule [25]. Interestingly, the bindings of neurologically active molecules such as BDNF and FGF2 are specific and DP-dependent [19]. BDNF requires polySia with DP greater than 12 [24] and FGF2 requires polySia with DP greater than 17 [25]. Another interesting finding of the binding property of polySia–NCAM is the interaction between polySia–NCAMs themselves. This was demonstrated by the surface plasmon resonance-based method [27] in which the polySia–NCAM synthesized by either of ST8SIA2 and ST8SIA4 was shown to be able to associate with polySia–NCAM synthesized by ST8SIA4, but not by ST8SIA2 [27]. These different types of functions are considered to be related to the differences in the quantity and quality of polySia [19,27]. Therefore, the function of polySia is reconsidered with the intact polySia structure that has not yet been well-dissected.

The relationship between polySia, or *ST8SIA2*, and mental disorders has been reported [19]. PolySia in diseased brains has been reported at abnormally low levels for schizophrenia [28] and high levels for bipolar disorder [29]. As the structural and functional analyses of polySia [1,27,30,31,32,33] have been established, the precise evaluations of these mutations and single nucleotide polymorphism (SNP)s of *ST8SIA2* that have been reported to have some relationship with schizophrenia and bipolar disorder could be performed [1,26,27,33,34]. For example, the SNP-7 (rs545681995) of *ST8SIA2* observed in a schizophrenia patient [35] resulted in a lower enzymatic activity, which led them to produce lower quality and quantity of polySia–NCAM than those of wild type [26,27,33]. In addition, polySia–NCAM synthesized by the SNP-7-derived enzyme showed lower binding properties for neurologically active molecules, such as BDNF, FGF2 and dopamine [26,27,33]. Based on these studies, it was demonstrated that reported mutations or SNPs of *ST8SIA2* showed impaired polySia in structures and functions [1]. Recently, not only genetic factors but also environmental factors, such as stress, have been found to change polySia expression in the brain region specifically [36]. In addition, chlorpromazine, an anti-psychotic medication, has also been found to change the polySia expression in the prefrontal cortex [37]. Therefore, it is hypothesized that regulated expression of polySia on polySia–NCAM at an appropriate location and timing are important, and that the impairments of normal polySia expression lead to a high risk of mental disorders [1,19]. To confirm this, it is important to detect and analyze polySia in the brain correctly. However, it remains difficult to fully elucidate the mechanisms of polySia–NCAM, because it is challenging to analyze long acidic glycans as they are. Even for HS and chondroitin sulfate (CS), which have been studied for a longer period of time than polySia, determining their precise structure has not yet been achieved.

Analyzing polySia derived from brain samples has been largely dependent on one method: immunostaining using antibodies towards polySia [1]. Previous studies have used antibodies whose precise specificities have not yet been confirmed. As polySia–NCAM has a complex and unstable structure [1] and the quantity and quality of polySia, which is related to some diseases, are very important [1,19,38]. It is necessary to compare detection methods using the same samples and to analyze polySia–NCAM using several different methods. Therefore, we performed several conventional and newly established methods and comprehensive analyses of polySia–NCAM derived from six different developmental stages. With this new approach, we identified new types of polySia–NCAM at each stage.

## 2. Results

### 2.1. Brain Size, Protein Amount, and Sialic Acid Amount

**Figure 1:**
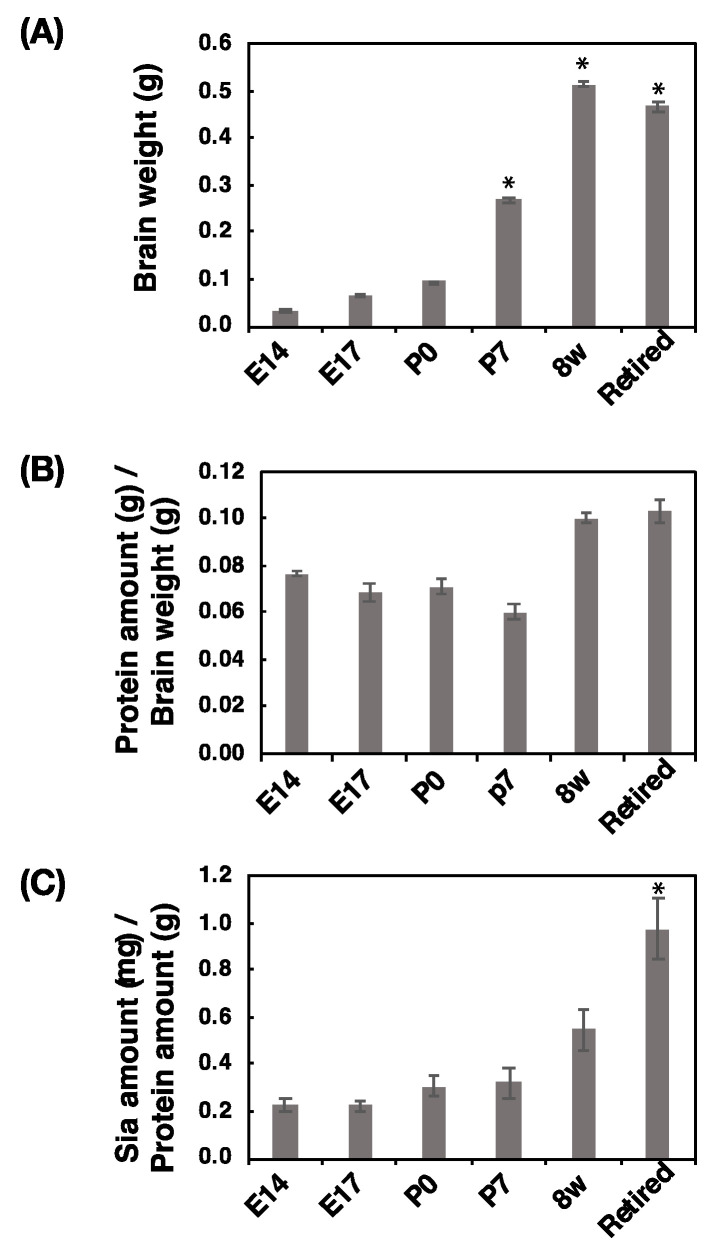
Comparison of brain weight, protein concentration, and Sia concentration at different developmental stages. Brains from six different developmental stages (E14, E17, P0, P7, 8 w, and retired (older than six months) (*n* = 3)) were used. (**A**) Comparison of brain weights of six different developmental stages. (**B**) Protein amount (g) per brain weight (g). Brains were homogenized using lysis buffer and a bicinchoninic acid (BCA) assay was performed to determine the protein concentration. (**C**) Sia amount (mg) per protein amount (g). Brain homogenate was treated with strong acid for hydrolysis of sialylglycoconjugates. All Sia was completely released and labelled with 1,2-dimethylenedioxybenzen (DMB). Sia–DMB were separated by a Wako Handy octadecylsilyl (ODS) column (250 × 4.6 mm, Wako). The absolute amount of Sia was calculated based on the authentic Neu5Ac. * *p* < 0.05.

The brain weight and amounts of protein and Sia of mouse developmental brains were measured. The brain weight of mice at 8 w was the heaviest among analyzed brains, and the brain weights increased from E14 to 8 w and decreased according to aging (Figure 1A). To determine the protein contents, we calculated the protein concentration (protein amounts/brain weight) and found that the values were almost the same among all tested individuals, although the adult brains were slightly richer in protein than embryonic and postnatal brains (Figure 1B). Then, we evaluated Sia contents by HPLC analysis and calculated the Sia concentration (Sia amounts/protein amounts) (Figure 1C). We found that the concentration of Sia increased according to the developmental stage, and in retired mice, the concentration was highest.

### 2.2. Western Blotting Analysis of PolySia–NCAM

#### 2.2.1. SDS–PAGE/Western Blotting Analysis

**Figure 2:**
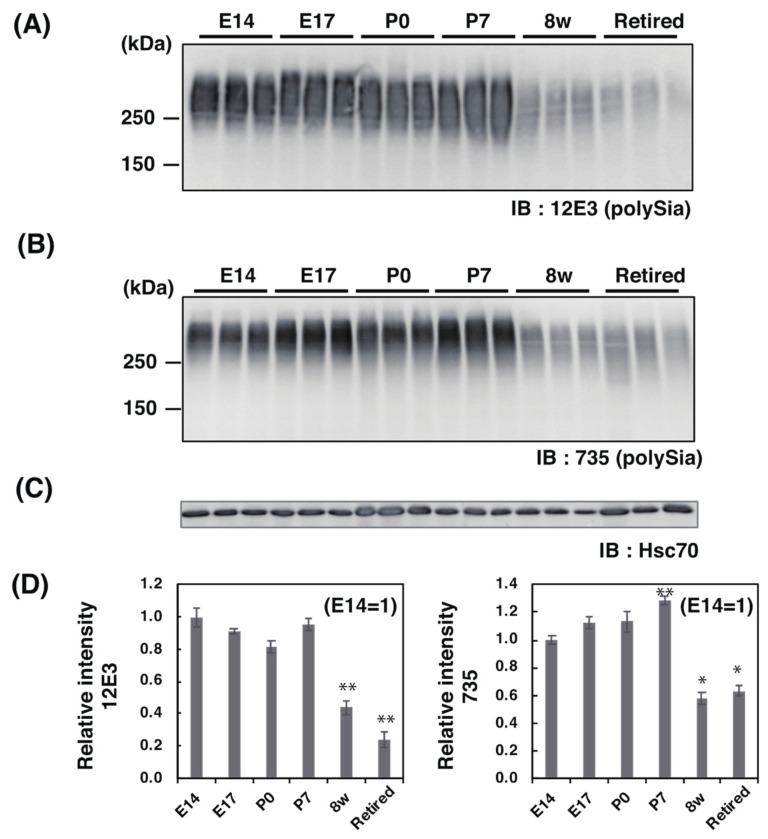
SDS–PAGE/Western blot analysis of polySia–NCAM derived from mouse brains at different developmental stages. PolySia expression at different brain development stages (E14, E17, P0, P7, 8 w, and retired, *n* = 3) was analyzed by SDS–PAGE/Western blotting. The brain homogenates were separated by SDS–PAGE (7% polyacrylamide gel) and blotted onto polyvinylidene difluoride (PVDF) membrane (10 μg as protein/lane). The polySia–NCAM was visualized by anti-polySia antibodies, (**A**) 12E3, and (**B**) 735. An anti-HSC70 blot was also used as the control (**C**). (**D**) The left panel shows the relative intensity of immunostaining of the 12E3 antibody. The right panel shows the relative intensity of the 735 antibody. E14 staining was set to 1.0. * *p* < 0.05. ** *p* < 0.01.

To compare the polySia in brains during brain development from E14 to retired, we first analyzed the polySia structure by the conventional SDS–PAGE/Western blotting using two defined anti-polySia antibodies (12E3 and 735) (Figure 2A–C). The molecular size of polySia staining was greater than 200 kDa, and the staining of the lower part was observed in adult brains (8 w and retired). 12E3 recognizes anti-oligo/polySia structure with non-reducing terminal-end, and 735 recognizes polySia structure in the internal polySia chains. The brain homogenates derived from E14 to P7 strongly showed the same levels of polySia staining (Figure 2D). On the other hand, polySia staining was drastically decreased to 40–50% until 8 w. In retired mice, the polySia levels were 25% evaluated by 12E3 and 60% by 735 (Figure 2D).

#### 2.2.2. Native PAGE/Western Blotting Analysis

**Figure 3:**
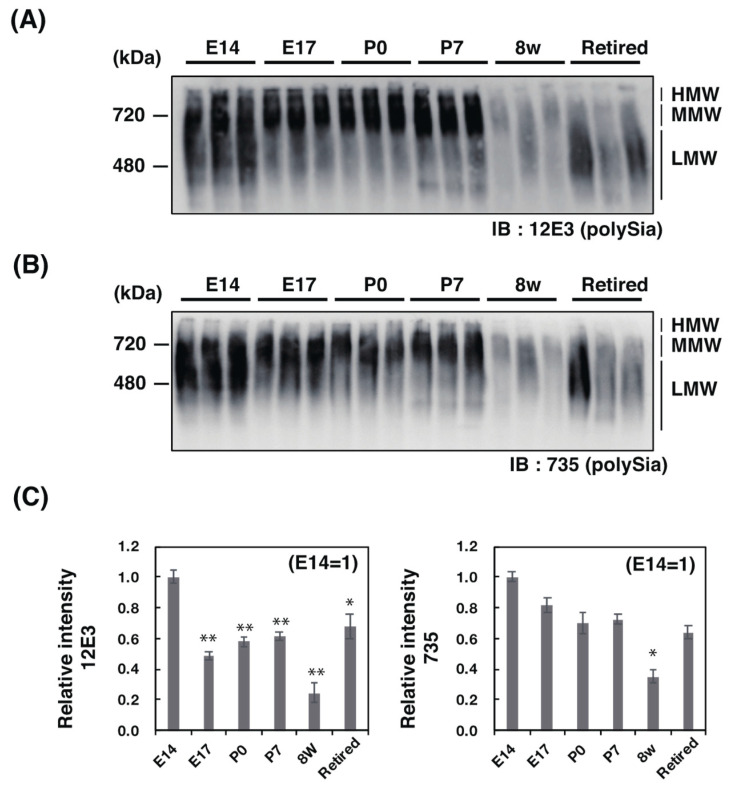
Native PAGE/Western blot analysis of polySia–NCAM derived from mouse brains at different developmental stages. PolySia expression at different brain development stages (E14, E17, P0, P7, 8 w, and retired, *n* = 3) were analyzed by native PAGE/Western blotting. The brain homogenates of six different stages were separated by native PAGE (7% polyacrylamide gel) and blotted onto polyvinylidene difluoride (PVDF) membrane (10 μg as protein/lane). The polySia–NCAM was visualized by anti-polySia antibodies, (**A**) 12E3 and (**B**) 735. (**C**) The left panel shows the relative intensity of immunostaining of the 12E3 antibody. The right panel shows the relative intensity of the 735 antibody. E14 staining was set to 1.0. * *p* < 0.05. ** *p* < 0.01.

SDS–PAGE analyzes denatured glycoproteins. As it is also important to understand the intact form of the molecule, information regarding whole molecular weight (Mw), charge, and conformation is required. Therefore, we selected the native PAGE for polySia analyses as, to our knowledge, no one has yet used this method for polySia analysis. Using native PAGE, we found strikingly different profiles of polySia–NCAM from those obtained by SDS–PAGE analysis (Figure 3A,B). The intensity of polySia staining was found to be highest at E14 before decreasing until P7 (Figure 3C). Staining derived from 8 w brain homogenate was the lowest (Figure 3C). Interestingly, polySia levels once again increased in retired mice (Figure 3C). Another interesting point is that the observed Mw differed between E14 and E17-P7 (Figure 3A,B). These different profiles were not possible to observe using SDS–PAGE (Figure 2A,B). These results indicate the presence of the different forms of polySia–NCAM if the sample was intact. There were three types of polySia–NCAM; the high Mw form (HMW, Mw above 720 kDa), middle Mw (MMW, around 720 kDa), and low Mw (LMW, lower than MMW) (see also Figure S2, lower panel). The HMW was only observed for 12E3 (Figure 3A). The HMW of polySia–NCAM in 8 w then changed to LMW (less than 480 kDa) in retired mice, although there were individual differences that appeared by native PAGE in the adult brain.

#### 2.2.3. SDS–PAGE/Native PAGE (S/N) MAP Analysis

**Figure 4:**
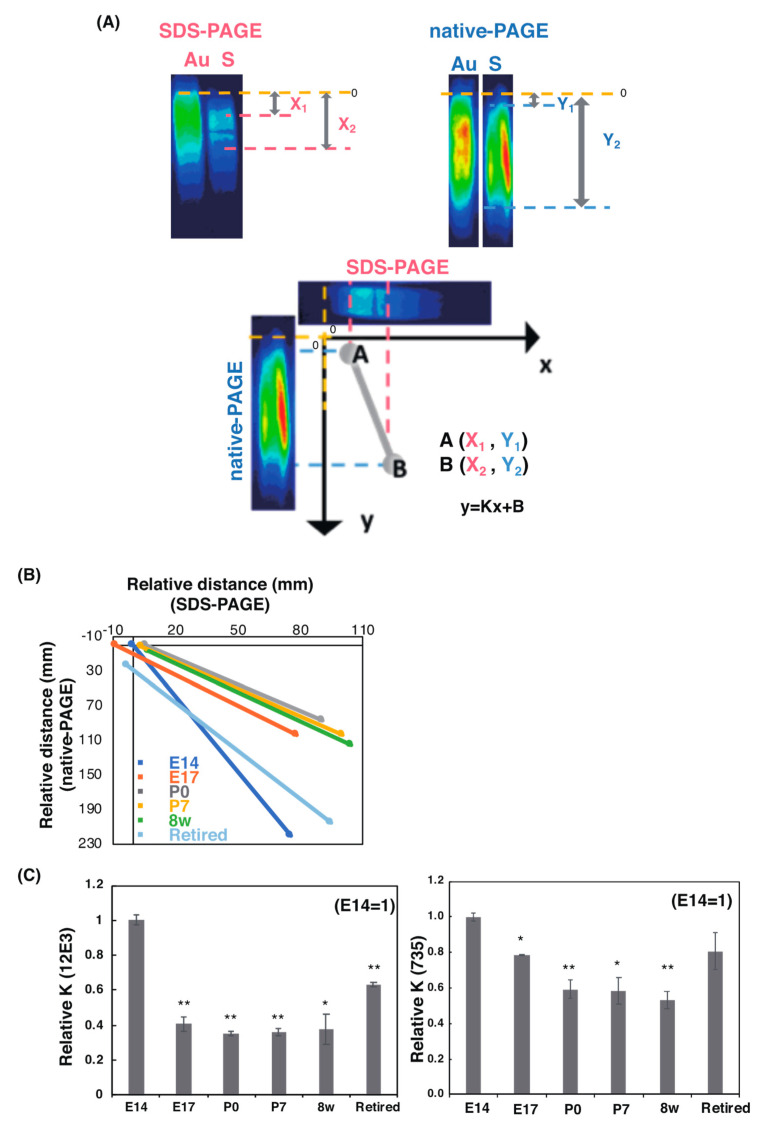
SDS-PAGE/Native PAGE MAP. The S/N MAP of brains at six different developmental stages was described based on the results of blots with each antibody. (**A**) The staining in Figure 2 and Figure 3 were changed to the pseudo color. The top of the staining of E14 was set as the origin (upper panel). The S/N map was obtained (lower panel) based on plot A (X_1_, Y_1_) and B (X_2_, Y_2_). Au: authentic sample; S: sample. (**B**) The S/N map of 12E3 staining (*n* = 1). The correlation curve of six different developmental brains is shown. (**C**) Relative slope value, K. The value of E14 was set to 1.0. The left panel shows 12E3 and the right panel shows 735. The values were obtained from the S/N maps from 6 different stage (*n* = 3). * *p* < 0.05. ** *p* < 0.01.

To understand the polySia profiles observed on the blots more critically, we established S/N Map analysis (Figure 4A). Using pseudo color of polySia staining obtained in Figure 2 and Figure 3 (Figure 4A, upper panel), we defined the migration (staining start and stop as plot A (X_1_, Y_1_) and B (X_2_, Y_2_) based on the authentic sample locations (E14 staining in this case)) of polySia using SDS–PAGE and native PAGE/Western blotting. Based on plots A and B, one line could be described and was shown as a formula: *y* = Kx + B (K indicates a slope) (Figure 4A, lower panel). With these values, we evaluated the quality of the whole polySia–NCAM by PAGE analysis (Figure 2 and Figure 3). Using these profiles, we found that the slopes of E14 and retired samples were completely different from those of E17 to 8 w (Figure 4B,C). The K value of E14 differed from those of E17 to 8 w. The K value of E17 to 8 w was almost the same, indicating that the profiles are similar, although the K value of E 17 obtained from 735 staining was different from that obtained from 12E3. The K value of the retired mice also showed different properties derived from native PAGE (Figure 3A,B). This property cannot be observed by conventional SDS–PAGE/Western blotting analysis.

#### 2.2.4. ELISA Analysis

**Figure 5:**
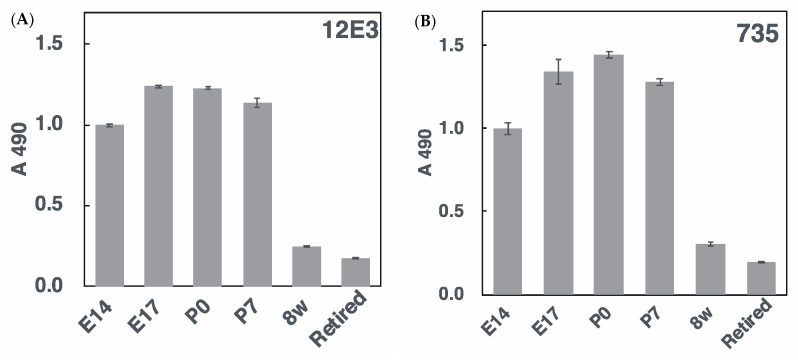
ELISA analysis of polySia–NCAM derived from mouse brains at different developmental stages. PolySia expression at different brain development stages (E14, E17, P0, P7, 8 w, and retired (*n* = 1)) was analyzed by ELISA. Brain homogenates of six different stages (250 ng as protein) were immobilized onto the 96-well plate and blocked with 2% BSA. The wells were then incubated with 12E3 or 735 antibodies before or after Endo-N treatments. After color development, the subtraction of the Endo-N-treated well from the Endo-N-untreated well was performed. (A) and (B) show the activity of the 12E3 and 735 antibodies, respectively. Triplicated analyses were shown with error bar.

To accurately determine the polySia amounts, we used the ELISA method in the presence and absence of endo-*N*-acylneuraminidase (Endo-N) treatments. As shown in Figure 5, the tendency was similar to, but not the same as, that of SDS–PAGE. Especially, the P7 sample showed different results. Even analyses of an intact sample showed different results (Figure 3 and Figure 5), indicating that the immobilization of polySia–NCAM onto the hydrophobic surface is different probably due to the different types of polySia–NCAMs.

### 2.3. ChemicalAanalysis of PolySia–NCAM

#### 2.3.1. Mild Acid Hydrolysis–Fluorometric Anion-Exchange Chromatography Method (MH–FAEC, Oligo-Analysis)

**Figure 6:**
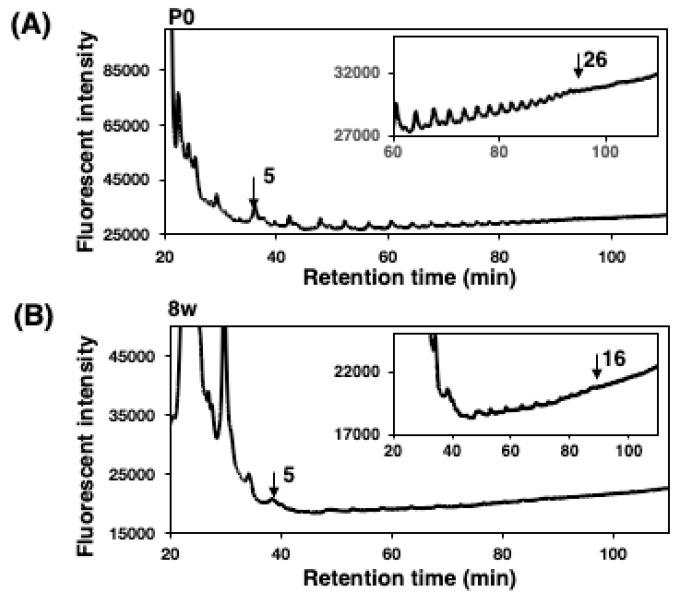
Mild acid hydrolysis–fluorometric anion-exchange chromatography analysis (oligo-analysis) of brain homogenates derived from mouse brains at different developmental stages (P0 and 8 w). Oligo/polySia analysis was applied for the detection of max. DP of the minimum polySia chain (polySia is easy to degrade). Brain homogenates (100 μg as protein) of P0 and 8 w were subjected to mild acid hydrolysis followed by DMB derivatization. DMB-labeled oligo/polySia chains were separated by an anion-exchange chromatography–HPLC analysis. Chromatogram of P0 (**A**) as an embryonic brain. Chromatogram of 8 w as an adult brain (**B**).

To understand the DP difference, we analyzed homogenates by the MH–FAEC method, originally established by us [1,31]. Based on this method, we observed the maximum DP of the polySia structure released under mild acid conditions, although the longest DP in the sample may not have been critically defined because of the instability of the polySia chain, even under mild acidic conditions [1,31,39]. We used two samples of brain homogenates, P0 as an embryonic form and 8 w as an adult form, in this method, and found that max. DP was 26 and 16, respectively (Figure 6A,B). These results indicate that the polySia chain derived from P0 is longer than that derived from adult 8 w with a difference of 10 DP. These results were consistent with those obtained using pig embryonic and adult brains [39].

#### 2.3.2. Fluorometric C_7_/C_9_ Analysis

**Figure 7:**
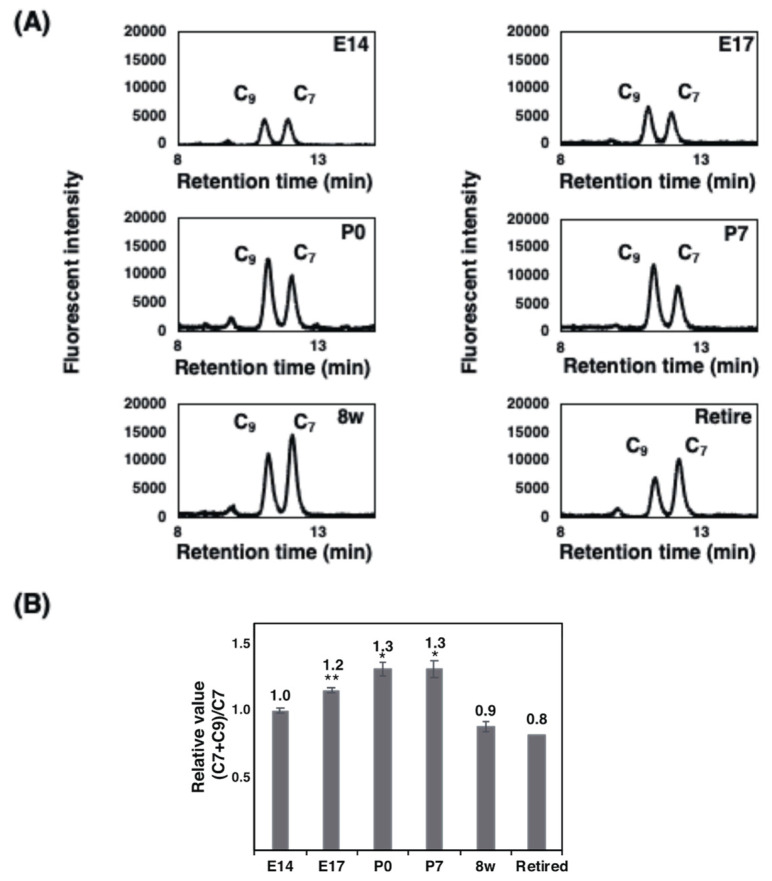
Fluorometric C_7_/C_9_ analysis of brain homogenates derived from mouse brains at different developmental stages. The fluorometric C_7_/C_9_ method was applied to analyze average DP. Brain homogenates (20 μg as protein) of six different developmental brains were used for the analysis (*n* = 3). **(A)** Typical chromatograms of C_7_/C_9_ analysis of each brains. (**B**) (C_7_ + C_9_)/C_7_ index in each brain. * *p* < 0.05. ** *p* < 0.01.

To determine the amounts of α2,8-linked polySia structure qualitatively, we performed fluorometric C_7_/C_9_ analyses, established by us [1,30]. Using this method, we evaluated the non-reducing terminal end of the sialic acid as the C_7_ analogue and α2,8-linked internal sialic acid as the C_9_ derivatives due to the mild periodate oxidation reaction [1,30]. In this experiment, we analyzed the samples pretreated with α2,3- and α2,6-sialiadse to remove the monoSia epitope, then blotted to the polyvinylidene difluoride (PVDF) membrane. We used the area over 100 kDa. Based on this method, the relative value of the estimated (C_7_ + C_9_)/C_7_ was increased from E14 to P7 (Figure 7A,B). As the max. DP of the minimum polySia at P0 was 26, E14 could be estimated as 20 and E17 as 24. In adults, the difference was 70%, indicating that the DP was 18, which is consistent with the results obtained in Figure 6 (8 w, 16).

### 2.4. Chromatographical Analysis of PolySia–NCAM

#### 2.4.1. Anion-Exchange Chromatography of PolySia–NCAM and NCAM

**Figure 8:**
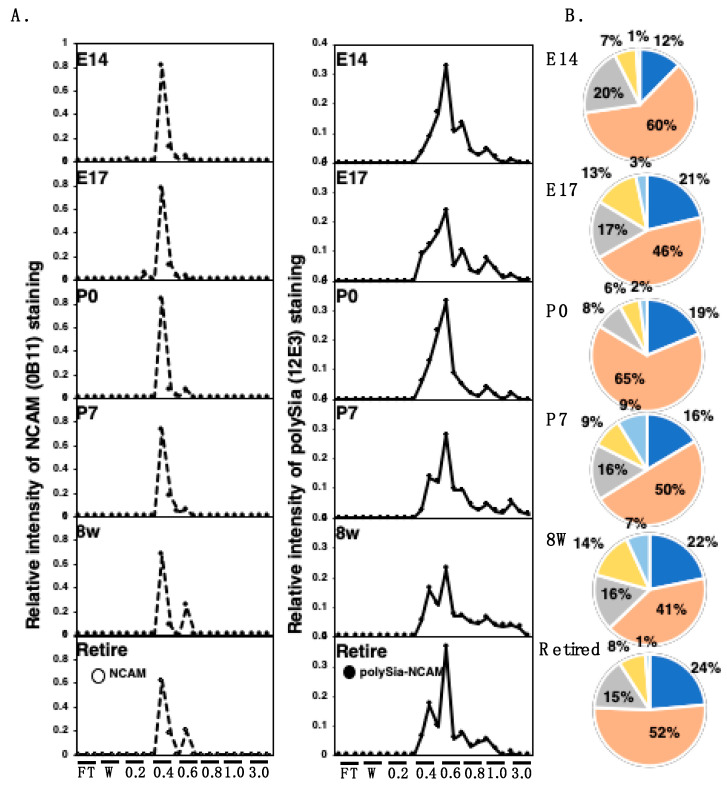
Chromatograms of polySia–NCAM and NCAM derived from mouse brains at different developmental stages analyzed by DEAE-Sephadex A-25 anion-exchange chromatography. (**A**) Elution profiles of anion-exchange chromatography of polySia–NCAM. Crude samples (solid line) or samples treated with Endo-N (broken line) were subjected to a DEAE-Sephadex A-25 anion-exchange column (500 μg as protein). The samples were step-wisely eluted with 1.5 mL of 2 mM Tris-HCl buffer containing 0.1% Triton and NaCl (0.2, 0.4, 0.6, 0.8, 1.0, and 3.0 M). Then, 10 μL of the sample from each fraction was analyzed by SDS–PAGE/Western blotting. For intact samples, most of the polySia on NCAM was detected by the anti-polySia antibody, 12E3 (right panel), while the Endo-N-treated sample, with only oligo-Sia–NCAM remaining, was detected by the anti-NCAM antibody (left panel). Flow-through (FT) fractions and wash buffer were composed of only 0.1% Triton and 2 mM Tris-HCl with 0.15 M NaCl. The sum of all fraction intensities detected by Western blotting was set to 1.0. (**B**) Comparison of the distribution of negative charges of the polySia–NCAM among brains at different developmental stages. The pie charts were described according to the chromatograms of anion-exchange chromatography of the crude sample. The intensity of 12E3 staining from each of the indicated NaCl concentrations was summed and the accumulation of the intensities of all lanes detected by Western blotting was set to 100%. Blue, pink, grey, yellow, and sky blue were 0.4 M, 0.6 M, 0.8 M, 1.0 M, and 3.0 M, respectively.

To determine the net negative charge (NNC) of polySia or polySia–NCAM, we applied each brain homogenate and endo-N-treated brain homogenate to anion-exchange chromatography. After loading the samples onto the DEAE-Sephadex A-25, we eluted the samples step-wisely using 0.2 M NaCl to 3 M NaCl solution with detergent. We monitored polySia elution by anti-polySia antibody (intact brain homogenate) and NCAM elution using an anti-NCAM antibody (Endo-N-treated homogenate) after SDS–PAGE/Western blotting for the eluted samples. The elution profiles observed using antibodies are shown in Figure S1 (polySia and NCAM). As shown in the chromatograms of the NCAM, the majority of NCAM elution was the same at 0.4 M (Figure 8, NCAM). At 8 w and retired, the NCAM elution at 0.6 M was also observed, indicating that the anionic NCAM was not due to the polySia being present in 8 w and retired brains, because the samples were treated with Endo-N. As for the polySia–NCAM elution (Figure 8, polySia), it began at 0.4 M and continued until 3 M. The ratio of the polySia–NCAM eluted at each concentration of NaCl is shown in the pie chart (Figure 8, polySia, right panel). The majority of polySia elution was 0.6 M, indicating that DP18 was present based on the calibrated elution position. This is consistent with the results shown in Figure 6. The elution of 0.4 M was estimated as DP = 5, 0.6 M as DP = 23, and 0.8 M as 140. Based on the results that the NCAM elution was almost 0.4 M and that polySia–NCAM was 0.6 M, the difference in the elution was found to be due to the Endo-N. Therefore, the majority of the polySia structure is estimated to be around 18. This value is again consistent with that of MH-FAEC (Figure 6). The 0.4 M elution that was considered to have low anionic residues increased according to development. The elution at 3.0 M NaCl was considered with a high charge, indicating that the complex formation, which cannot be separated under detergent, increased until P7 and continued at 8 w but dramatically decreased in retired mice. The sum of 0.6 M to 1.0 M was almost the same (approximately 80~90), although E14 had the highest content. The estimated NNC, taking into account the ratio, is summarized in Table S1.

#### 2.4.2. Gel Filtration

**Figure 9:**
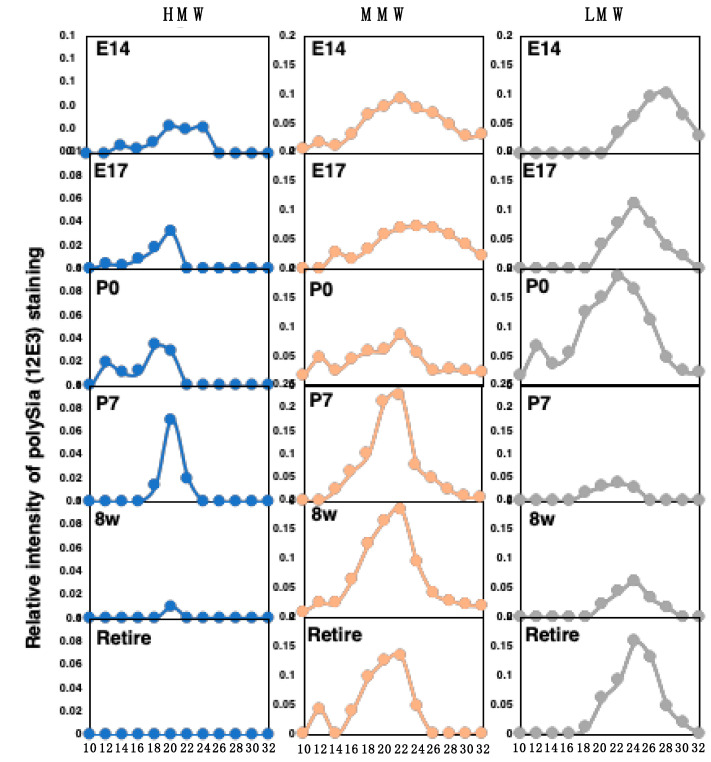
Gel filtration of polySia–NCAM derived from mouse brains at different developmental stages. Brain homogenates were analyzed by Sephacryl S-500 gel filtration chromatography. The relative intensity was calculated by the 12E3 staining of native PAGE, as shown in Figure S2. The staining was categorized into three molecular species: HMW (above 720 kDa), MMW (480–720 kDa), and LMW (less than 480 kDa), according to the staining area. The sum of the intensity of the immunostaining of the fractions was set to 1.0.

To determine the Mw of the intact polySia–NCAM, as it was predicted to form a complex (Figure 8, 0.8–3 M elution), we performed gel filtration using Sephacryl S-500 chromatography. The fractions at 20, 22, and 24 were calibrated as 2000, 510, and 130 kDa, respectively. We also performed native PAGE analysis for every two fractions (Figure S2). Based on the native PAGE, we identified three areas: the HMW (above 720 kDa), MMW (720~480 kDa), and LMW (less than 480 kDa) areas (Figure S2). The elution profile based on native PAGE analyses is shown in Figure 9. The HMW of E14 observed by native PAGE was eluted at Fr. 22-24 and a small amount at Fr. 14. On the other hand, the HMW of E17 to P7 was eluted at 12–20, indicating that the types of complex formation differed. At the MMW, the peak position of the broad elution of E14 and E17 was Fr. 24, while that of P0 to retire was 22. The LMW of E17 was eluted at 28, while others were eluted at 24. All data are summarized in Table S2. These results demonstrate that embryonic and postnatal brains (E14 to P7) have high molecular species levels of polySia–NCAM compared to adult brains (8 w and retired).

## 3. Discussion

In this study, we analyzed polySia by conventional and new biochemical and chemical methods using mouse developmental brains (E14, E17, P0, P7, 8 w, and retired) and performed comprehensive analyses of polySia on polySia–NCAM for quantity and quality. The methods used in this study are summarized in Table 1.

For Western blotting, we used two different types of anti-polySia antibody: 12E3 and 735. 12E3 recognizes the polySia structure with a non-reducing terminal end at DP ≥ 5 [40], and 735 recognizes the internal polySia structure at DP ≥ 11 [32,41]. The quantity of polySia from the brain homogenates observed by SDS–PAGE/Western blotting was almost the same when evaluated with 12E3 (Figure 2D, left). On the other hand, band staining increased from E14 to P7 when evaluated by 735 (Figure 2D, right), indicating that the non-reducing terminal end of polySia was not changed, but the internal polySia structure was increased as estimated with the total sample. This observation was supported by the fluorometric C_7_/C_9_ analysis that can evaluate the internal α2,8-linked oligo/polySia amounts chemically (Figure 7B) [1,30]. It should be noted that the results of fluorometric C_7_/C_9_ analysis also include the contributions of the di-Sia to oligo-Sia structures, although only the region larger than 100 kDa on the PVDF membrane, in which the 12E3 and 735 epitopes were detected, was analyzed to minimize those contributions [32]. In 8 w brains, polySia was drastically decreased to approximately half (Figure 2D). In retired mice, the non-reducing terminal end of polySia was further decreased (Figure 2D, left), indicating that the polySia chain was reduced in retired mice, although the polySia observed by 735 was the same level as that of 8 w (Figure 2D, right). The difference might be due to the DP = 5–10 oligo/polySia chain that can be observed by 12E3 but not by 735. The decrease in polySia amounts from the embryonic to adult brain was the same result as that of pig brains [39]. The Mw of polySia–NCAM observed by SDS–PAGE was above 200 kDa, and these bands disappeared after Endo-N treatment, indicating that the staining was due to oligo/polySia (DP ≥ 5). As polySia staining was almost absent for NCAM-KO mice, the stain was due to polySia–NCAM.

Using native PAGE, we identified a new discovery of polySia. The migration of polySia–NCAM derived from E14 was completely different from that of E17, P0, P7, and 8 w (Figure 3A,B). In retired mice, the staining patterns were again completely different (Figure 3A,B) from others, indicating that polySia–NCAM forms several different complex types, probably due to the binding to different types of molecules as the SDS–PAGE pattern of polySia–NCAM was almost the same (Figure 2A,B). The second interesting feature of polySia–NCAM observed by native PAGE was the staining intensity. Particularly in retired mice, the polySia–NCAM staining was stronger than that of SDS–PAGE with an unknown mechanism. In E14, complex formation above 720 kDa was only observed by 12E3, indicating that polySia–NCAM in HMW might be due to the polySia–NCAM with DP 5~10. The separation of MMW and LMW was observed in E14. In retired mice, only LMW was observed, indicating that LMW might be one of the markers for aging. Among E17 to P7, the complex was almost the same and present as MMW. This form might be important for the neuronal network formation, and the LMW observed in E14 might be important for neurogenesis, and thus, it is important for the identification of the complex. For E14 to P7, the staining of polySia is uniform among the mice used; however, the polySia staining pattern was slightly different in 8 w and retired mice, indicating that there was a difference among mice used, probably due to the environmental conditions for each mouse.

To better show the differences, we developed a SDS–PAGE/native PAGE map (Figure 4). With this profile, we can show the different quality of polySia–NCAM in each brain by slope value. The slope of E17, P0, P7, and 8 w was almost the same, and interestingly, the slopes of E14 and retired were completely different, indicating that the types of polySia–NCAM in E14, E17–8 w, and retired are different. Therefore, this map is powerful for showing the different quality of molecules. The cause of the different pattern of polySia–NCAM in native PAGE was considered to be charge, Mw (complex formation), or conformation of polySia–NCAM in each brain. To confirm this, we analyzed NNC of polySia–NCAM using anion-exchange chromatography. The average NNC was calculated based on the elution position and ratio of the samples and is shown in Table S1. The NNC at P7 and 8 w, was approximately 150–200 (0.8–3.0 M components for polySia–NCAM in Figure 8A right); however, the DP estimated by oligo-analysis, and C_7_/C_9_ analysis showed that 8 w was 10% shorter than that of P7 and P0, and the DP of P0 was 26, indicating that the higher NNC might be due to the strongly binding acidic molecules, such as glycosaminoglycan, and such components might be rich in P7 and 8 w. PolySia–NCAM forms a specific complex with such components that cannot be released by anion-exchange chromatography with detergent. Interestingly, the NNC was dramatically decreased for retired mice, showing that the presence of polySia–NCAM changes during aging, although the high-charge NCAM observed in the adult brain did not change (0.8–1.0 M components). The 0.2 M change of NCAM observed for 0.4 to 0.6 M NCAM components (Figure 8A left) might be due to the additional sulfation, as observed in chick brains [42]. Because the negative charge of the carboxyl group (pKa = 4.76) is weaker than that of the sulfate group (pKa = 1.96), the 0.2 M difference (0.4 to 0.6 M difference, corresponding to DP of polySia around 20) might be explained by the presence and absence of sulfate groups.

Then, we analyzed gel filtration profiles to determine the molecular size to understand the possibility of complex formation. We estimated the size of polySia–NCAM observed by native PAGE and gel filtration. The HMW was estimated as 2000 kDa, indicating the complex formation of polySia–NCAM with other components. The HMW was observed in E14 to P7 but was richest for P0. Therefore, the high charge might be due to the HMW, especially in P7. In 8 w, the component decreased to 1%, and in retired mice we did not observe the component, indicating that the HMW component might be the important polySia–NCAM complex form for neurogenesis. LMW is estimated as 150~200 kDa, indicating the monomer type of polySia–NCAM. An interesting point is that even for monomer-type polySia–NCAM, the elution position was different in the case of E14. The monomer of polySia–NCAM derived from E14 is different from that of other polySia–NCAMs, probably due to the epitope that cannot form a complex with polySia–NCAM or other components. The MMW is estimated as ~500 kDa, indicating the polySia–NCAM dimer form or complex type of polySia–NCAM with other components (Mw around 250 kDa). The broad peaks derived from E14, E17, and P0 differed from those of sharp peaks of P7, 8 w, and retired, indicating that associated components might be different. Proteoglycans, such as HSPG, CSPG, or other specific forms, are considered to associate with polySia–NCAM based on the elution profile of anion-exchange chromatography. As the Ig2 domain has been found to bind to HS and CS [1], although nobody has yet assessed the possibility with intact samples to the best of our knowledge, it would be interesting to investigate the possibility of a glycosaminoglycan–polySia–NCAM interaction, and we are now searching for the complex. In addition, BDNF and FGF2 could also be involved in the complex formation with polySia–NCAM, because it was proven that polySia forms large complexes (2000~5000kD) with BDNF and FGF2 [1]. Other polySia binding molecules in the brain, such as histone H1 [43] and myristoylated alanine-rich C kinase [44], might also be involved in the formation of the complex observed in this study.

**Figure 10:**
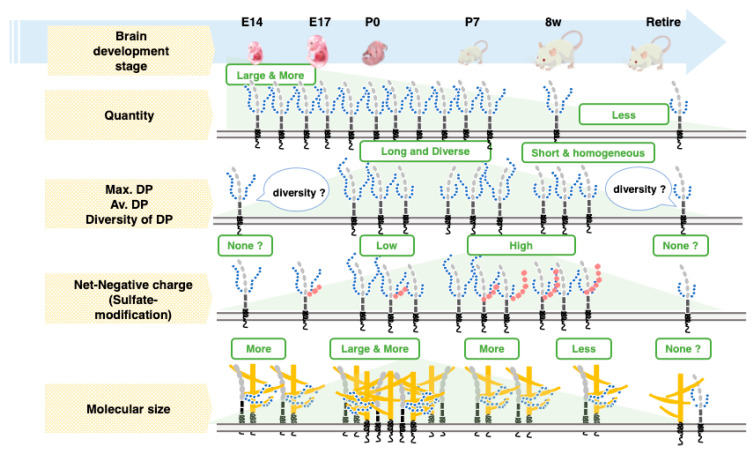
The quantity and quality of polySia and features of polySia–NCAM in brain development. polySia–NCAM is highly regulated in both quantity and quality (DP, net negative charge, and size) during brain development. The quantity of polySia–NCAM is decreased from E14 until retired, although re-increased in retired mice in the native PAGE technique. The DP of polySia is postnatally decreased during development; however, the net negative charge and molecular size of polySia are not always consistent with the change of DP but appear to be unique to the developmental stages.

Brain sizes increased depending on development, and the retired mice showed smaller sizes than 8 w mice, despite the protein concentration being the same between 8 w and retired mice. Interestingly, the amount of Sia increased depending on the development, and for the retired mice, the contents of Sia were at the maximum. By calculating the ratio (polySia/Sia), E14 was found to be the highest and lowest at 8 w. The retired mice showed a slight increase. Therefore, again, polySia–NCAM expression was found to decrease from E14 to adult and increase during aging. The effective expression of polySia at E14 might be important. Based on the comprehensive analysis, we summarized the polySia–NCAM during development in Figure 10.

PolySia or polySia–NCAM is reported to have a relationship with mental disorders, such as schizophrenia, bipolar disorder, and autism. The gene for a biosynthetic enzyme of polySia, *ST8SIA2*, actually contains mutations or significant SNPs that are statistically associated with mental disorders, e.g., SNP-7, rs3759916, and rs3759914 from schizophrenia patients in a Japanese population [35] and rs2168351 from bipolar disorder patients [45]. These statistical data suggest that the identified SNPs in *ST8SIA2* are a genetic factor of mental disorders. However, interestingly and most importantly, those SNPs are unambiguously demonstrated to bring about impairments of structures and functions of polySia, as far as evaluated by biochemical and functional analyses [1,19,26,27,33,34,46]. In addition, environmental factors that significantly increase the risk of pathogenesis of mental disorders also influence the expression profile of polySia–NCAM in a brain-region-specific manner [36,37]. Thus, both genetic and environmental factors of mental disorders greatly affect the structure and location of polySia–NCAM in the brain. Based on these studies, it is hypothesized that the expression of normal polySia at the appropriate location and timing is important, and that any impaired features of expression may cause mental disorders [19]. To validate this hypothesis, it is important to demonstrate what the normal polySia–NCAM is. So far, estimation of polySia structure has almost always been conducted by SDS–PAGE/Western blotting using appropriate or sometimes inappropriate anti-polySia antibodies. Although some studies have used several chemical methods, more effective methods are necessary for the critical diagnosis of mental disorders and other diseases, because we already understand, as the present study demonstrates, that there are several forms of polySia–NCAM in terms of quality. It is important to understand polySia in terms of quantity and quality toward samples for analysis and diagnosis by comprehensive aspects of the polySia.

## 4. Materials and Methods

### 4.1. Materials

Bovine serum albumin (BSA), anti-NCAM antibody (OB11), α2-3,6-sialidase from *Clostridium perfringens* (Recombinant, *E. coli*, Cat. No. 480708), and trifluoroacetic acid (TFA) were purchased from Sigma-Aldrich (Missouri, MO, USA). The anti-polySia antibody, 12E3, which recognizes the oligo/polyNeu5Ac structure (DP ≥ 5) [40], was generously provided by Dr. Tatsunori Seki (Tokyo Medical University, Japan). 735-ScFv, which recognizes the polyNeu5Ac structure (DP ≥ 11) [32], was purified, as described previously [41]. Endo-N, which cleaves the oligo/polySia structure (DP ≥ 5) [47], was generously provided by Dr. Frederic A. Troy (University of California, Davis, CA, USA). POD-labeled anti-mouse IgG + IgM and anti-rabbit IgG were purchased from American Qualex (San Clemente, CA, USA). Colominic acid, α2,8-linked polyNeu5Ac (average DP = 43), which is chemically and immunologically identical to the polySia structure in NCAM and phenylmethylsulfonyl fluoride, was purchased from Wako (Osaka, Japan). The PVDF membrane was obtained from Millipore (Burlington, MA, USA). Enhanced Chemiluminescence Western blotting Detection Reagent, Sephacryl S-500, and DEAE-Sephadex A-25 were obtained from GE Healthcare (Buckinghamshire, UK). 1,2-dimethylenedioxybenzen (DMB) was purchased from Dojindo Molecular Technologies, Inc. (Kumamoto, Japan). Pre-stained Mw marker was obtained from Bio-Rad (Hercules, CA, USA). The nti-HSC70 antibody, NativeMark Unstained Protein standard, and BCA protein assay reagents were obtained from Thermo Fisher Scientific (Waltham, MA, USA).

### 4.2. Animals and Ethics Statement

Mice (*Mus musculus*, C57/BL6J, E14, E17, P0, P7, 8 w, and retired (at least six months)) were maintained in a controlled environment (23 ± 2 °C and 50 ± 10% humidity, 12:12 light/dark cycle) with food and water available ad libitum. All procedures were approved by the Animal Care and Use Committee of Nagoya University (Permit Number: 2016022506, Permit date: 31 March 2016). We have adopted the 3R principles (replacement, reduction, and refinement) and test plans were formulated with maximum consideration for minimizing pain on the requisite minimum number of animals, with respect for the lives of the animals, taking animal welfare into consideration. We used the minimum animal numbers (*n* = 3) necessary for statistical analysis.

### 4.3. Sample Preparation

All brains were collected by surgery. The brains were homogenized with lysis buffer (1% Triton X-100, 1 mM phenylmethanesulfonyl fluoride, protease inhibitors: 1 μg/mL aprotinin, 1 μg/mL leupeptin, 1 μg/mL pepstatin, 2 μg/mL antipain, and 10 μg/mL benzamidine), 1 mM EDTA, 50 mM NaF, 10 mM β-glycerophosphate, 10 mM sodium pyrophosphate, and 1 mM sodium *o*-vanadate in phosphate buffered saline (PBS). The homogenates were incubated on ice for 1 h and centrifuged at 9600× *g* for 15 min at 4 °C. The supernatant was collected. Protein concentrations were measured using the BCA assay.

### 4.4. Content of Sialic Acid

Sia was measured as previously described [1,30]. Briefly, samples (25 μg protein as BSA) were diluted with an equal volume of PBS and hydrolyzed with 0.1 NTFA at 80 °C for 2 h. The samples were then dried by speed vac. To the dried samples, equal volumes of 0.01 NTFA and DMB solution were added and incubated at 50 °C for 2 h. The authentic Neu5Ac sample was used for evaluation. DMB derivatives were separated by the Wako Handy ODS column (250 × 4.6 mm, Wako, Osaka, Japan).

### 4.5. Western Blotting

First, 10 μg of protein from each sample was separated by SDS–PAGE (7% acrylamide gel) or native PAGE (7% acrylamide gel), and proteins were blotted on a PVDF membrane. The membrane was then blocked with 1% BSA with PBS containing 0.05% Tween 20 (PBST) at 25 °C for 1 h for anti-glycan antibody or 2% skim milk for anti-protein antibody. The membrane was incubated with the primary antibody. After washing with PBST, the membrane was incubated with the secondary antibody, peroxidase-conjugated anti-mouse IgG + M antibody (1/5000 dilution) at 37 °C for 1 h. Primary antibodies used were anti-polySia antibody; 12E3 (1.0 μg/mL, mouse IgM), 735 (0.8 μg/mL, mouse IgG), anti-HSC70 antibody (0.28 μg/ mL, rabbit IgG), and anti-NCAM antibody (0B11). This antibody can recognize 140 and 180 kDa types of NCAM [48].

### 4.6. SDS–PAGE/Native PAGE Map

To make the SDS–PAGE/Native PAGE MAP, first, polySia-staining obtained in SDS–PAGE or Native PAGE/Western was changed to pseudo color, and standard band was set as the origin of the map (in this case, E14 mouse brain homogenate). Then, the distance from the origin to the top of the other band on SDS–PAGE (X_1_), and that on Native PAGE (Y_1_) was calculated. Additionally, the distance from the origin to the bottom of the other band on SDS–PAGE (X_2_), and that on Native PAGE (Y_2_) was calculated. Two points, A (X_1_, Y_1_) and B (X_2_, Y_2_), were plotted on a coordinate axis and linked with each other to make a correlation curve, *y* = K x + B. All curves from different samples were plotted on the same map, and the slope (K) can be compared to reflect the qualitative differences of the polySia from each sample.

### 4.7. ELISA Analysis

Samples were adjusted to 5 μg/mL (as BSA) with PBS (the concentration of Triton X-100 should be below 0.03%), and 50 μL of the solutions was serially diluted and absorbed onto a 96-well immunoplate, and other procedures were performed as described [39].

### 4.8. Mild Acid Hydrolysis–Fluorometric Anion-Exchange Chromatography Analysis

Samples (1.0 mg protein as BSA) in 200 μl of 0.005N TFA were added to 200 μL of DMB solution and incubated at 50 °C for 1 h [1,31,39]. Then, cold ethanol was added to the samples (final concentration, 95%) and stood at −80 °C for 2 h. After centrifugation, the supernatants were dried by speed vac. The dried samples were dissolved in water and NaOH was added to a final concentration of 0.1 N, and the solution was then incubated at 37 °C to remove lactonization. After neutralization with 1 N HCl, the samples were diluted and subjected to an anion-exchange chromatography column (DNApac PA-100, 4 × 250 mm, DIONEX) and separated on a JASCO HPLC system. The sample was loaded on a column and eluted with 2 mM Tris-HCl (pH 8.0), followed by NaCl gradient (0–15 min, 0 M; 15–185 min, 0–0.6 M; 185–190 min, 0.6–1.0 M) in 2 mM Tris-HCl (pH 8.0) and 0.1% triton X-100. The flow rate was 1 mL/min, and fractions were monitored with a fluorescent detector (Em 373 nm, Ex 448 nm, FP-2020, JASCO, Tokyo, Japan).

### 4.9. Chemical Analysis of α2,8-Linked Oligo/PolySia Chains on Glycoproteins Blotted onto PVDF Membranes

Brain glycoproteins (100 μg protein as BSA) in 14 μL were added to 4 μL of 5 × reaction buffer and 2 μL of α2-(3,6)-sialidase treatment (25 μU) and incubated at 37 °C for 1 h to release monoSia residues. The sialidase-treated samples were blotted onto PVDF membranes, as described previously, and areas above 100 kDa were cut out. The membranes were analyzed by the fluorometric C_7_/C_9_ method for internal sialyl residues, as previously described [1,30].

### 4.10. Analysis of PolySia–NCAM from Brain Homogenates Using Anion-Exchange Chromatography

Untreated or Endo-N-treated brain samples (500 μg BSA) were applied to DEAE Sephadex A-25 anion exchange chromatography (1 mL) and FT with PBS containing 0.1% triton X-100 was obtained. Then, samples were eluted with the 0.2, 0.4, 0.6, 0.8, 1.0, and 3.0 M NaCl in 2 mM Tris-HCl (pH 8.0) with 0.1% triton X-100. After the samples were electrophoresed with SDS–PAGE and blotted onto a PVDF membrane, the amounts of polySia and NCAM in each fraction were determined by anti-polySia staining (12E3) or anti-NCAM antibody staining of the membrane. The Endo-N-treated sample for NCAM staining and intact sample for polySia staining were used. The column was calibrated with colominic acid (authentic polySia).

### 4.11. Analysis of PolySia–NCAM from Brain Homogenates Using Gel-Filtration Chromatography

Intact brain samples (2 mg of the sample) were applied onto the Sephacryl S-500 (14 mL) and eluted with PBS containing 0.1% triton X-100. After the samples were electrophoresed with native PAGE and blotted onto a PVDF membrane, the amounts of polySia in each fraction were determined by anti-polySia staining (12E3). The column was calibrated with NativeMark Unstained Protein Standard.

### 4.12. Data Analysis

All values were expressed as the mean ± SE (n is three) and p-values were evaluated by the Student’s *t*-test in Figure 1, Figure 2, Figure 3 and Figure 4 and Figure 7. The value of each stage was compared with that of E14.

## 5. Conclusions

We analyzed polySia–NCAM by several conventional and newly developed chemical and immunochemical methods and found that polySia–NCAM showed different species during development (Figure 10). Therefore, it is important to evaluate polySia using appropriate methods, because widely used SDS–PAGE/Western blotting with one antibody is only one aspect of the polySia–NCAM present in the brain.

## Figures and Tables

**Table 1:** Methods for the evaluation of polySia–NCAM.

Name of Methods	Abbreviation Name	Quality	Quantity	Figure	References
SDS–PAGE/Western blotting	d-Western	+ * (MW of denaturated polySia–NCAM)	+	Figure 2	[1,19], Many, This paper
Native PAGE/Western-blotting	n-Western	+++ (Intact MW charge, conformation)	+	Figure 3	This paper
SDS-PAGE/native PAGE map	S/N map	+++	−	Figure 4	This paper
ELISA	ELISA	+ *	+	Figure 5	[1,19,39], This paper
MH–FAEC	Oligo-analysis	++ (minimum DP of the sample)	+	Figure 6	[1,27,31,34,39], This paper
Fluorometric C_7_/C_9_ analysis	C_7_/C_9_ analysis	++ (Estimated DP, average chain length)	++ (amounts of α2,8-linked Sia)	Figure 7	[1,26,30,32,36], This paper
Anion-exhange chromatography	NNC-analysis	+++ (Net negative charge of intact polySia–NCAM)	+	Figure 8	[26,27], This paper
Gel filtration	Size-analysis	+++ (observed MW of intact polySia–NCAM)	+	Figure 9	This paper

* The specificity of the used antibody contains the information of the quality.

## Supplementary Materials

Supplementary materials can be found at https://www.mdpi.com/1422-0067/21/16/5892/s1. **Figure S1.** The Western blot result of anion-exchange chromatography of polySia–NCAM derived from mouse brains at different developmental stages. **Table S1.** The average net negative charge of polySia-NCAM estimated by the anion exchange chromatography. **Figure S2.** Native PAGE/Western blot analysis of gel filtration chromatography of polySia–NCAM derived from mouse brains at different developmental stages. **Table S2.** The size of intact polySia-NCAM estimated by the native PAGE and gel filtration.

## Abbreviations

DP	Degree of polymerization
FGF	Fibroblast growth factor
FGFR	Fibroblast growth factor receptor
GAG	Glycosaminoglycan
HPLC	High performance liquid chromatography
Ig	Immunoglobulin
IgG	Immunoglobulin G
IgM	Immunoglobulin M
KO	Knockout
NCAM	Neural cell adhesion molecule
PAGE	Polyacrylamide gel electrophoresis
polySia	Polysialic acid
polySia–NCAM	Polysialylated neural cell adhesion molecule
Sia	Sialic acid
SNPs	Single nucleotide polymorphisms
