# Supplementary material for: Combinational Analyses with Multiple Methods Reveal the Existence of Several Forms of Polysialylated Neural Cell Adhesion Molecule in Mouse Developing Brains
Source: Int J Mol Sci. 2020 Aug 16;21(16):5892. doi: 10.3390/ijms21165892 (PMC7460633; doi:10.3390/ijms21165892)
Supplement: Supplementary file 1 [file ijms-21-05892-s001.pdf]

International Journal of *Molecular Sciences*

MDPI

Supplementary Materials

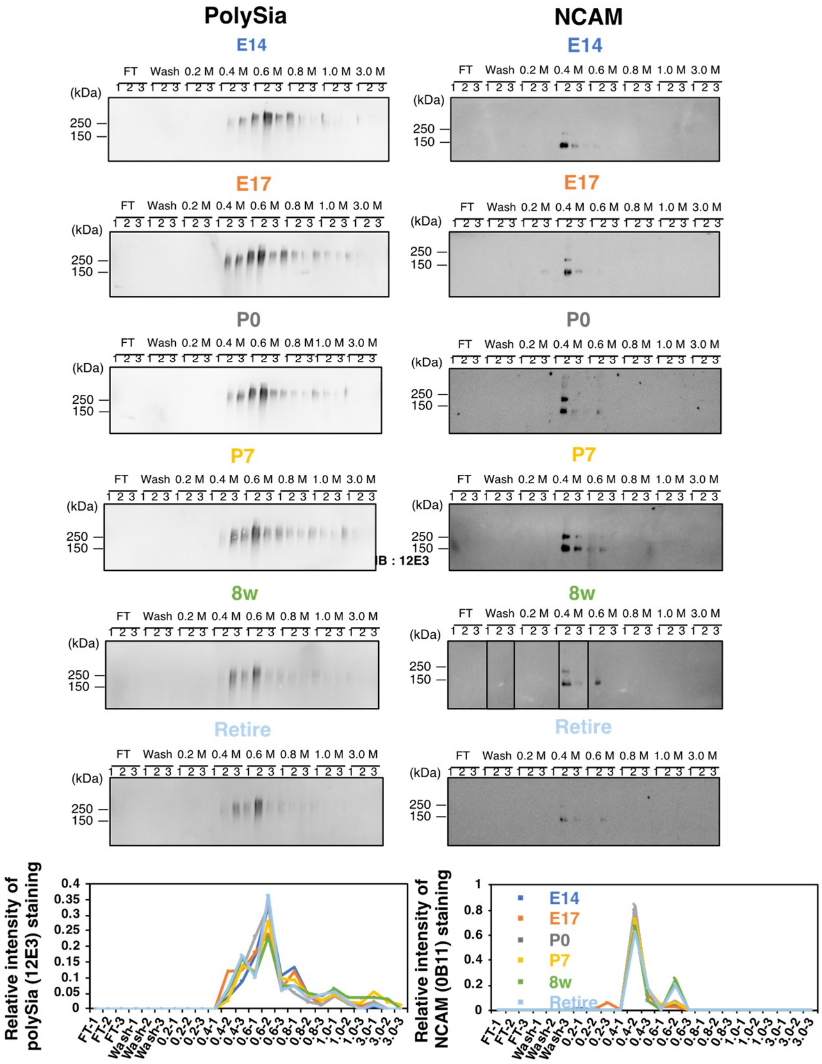

Figure S1. The Western blot result of anion-exchange chromatography. (**A**) The polySia-NCAM staining of eluted fractions. Samples from the anion-exchange chromatography fractions (10 µL) were analyzed by SDS-PAGE Western blot analysis to detect polySia (Left panel) or NCAM (Right panel). Left panel shows the immunoblots of anti-polySia antibody (12E3) in different vertebrate brains. Right panel shows the NCAM staining using 0B11. (**B**) An overlay of the chromatogram shown in Figure 6 (left panel, polySia-NCAM). An overlay of the chromatogram shown in Figure 6 (right panel, NCAM).

Table S1. The average net negative charge of polySia-NCAM estimated by the anion exchange chromatography

States	E14	E17	P0	P7	8W	Re
Average negative charge (M)	0.67	0.71	0.65	0.85	0.81	0.64
Estimated average DP	43	62	36	218	152	33

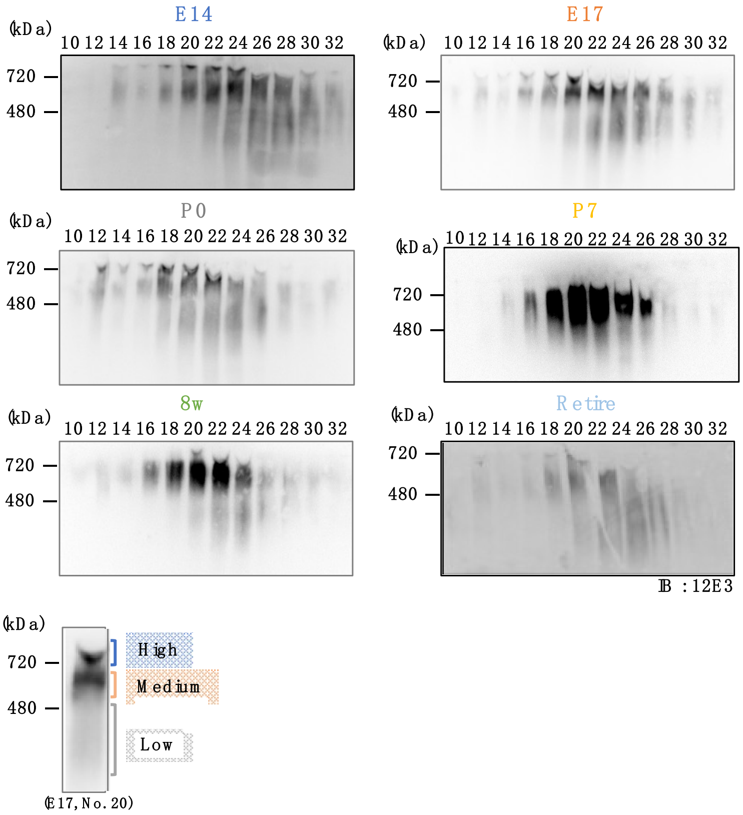

Figure S2. Native PAGE/Western blot analysis of gel filtration chromatography of polySia-NCAM derived from mouse brains at different developmental stages. The molecular size of polySia-NCAM was analyzed by gel filtration chromatography. Brain homogenates (2 mg as protein) were subjected to Sephacryl S-500 (15 × 310 mm) gel-filtration chromatography and fractions were collected in the same way as Figure 9. According to the molecular size, the staining was sorted to 3 types of different molecular species. High, medium, and low represent the oligomer, dimer, monomer of poySia-NCAM, respectively.

Table S2. The size of intact polySia-NCAM estimated by the native PAGE and gel filtration.

Type	MW (Native-PAGE, kDa)	MW (Gel Filtration, kDa)	Molecular Form of PolySia-NCAM Estimated
HMW	Over 720 kDa	2000	Homo/hetero-Oligomer
MWW	480–720 kDa	510	Homo/hetero-Dimer
LMW	Under 480 kDa	130	Monomer
